# FBS26: An Entropy-Based Metric for Assessing Architectural Maturity of UML Class Diagrams

**DOI:** 10.3390/e28101126

**Published:** 2026-10-14

**Authors:** Wiktor Bohdan Daszczuk, Stanisław Jerzy Niepostyn

**Affiliations:** 1 Institute of Computer Science, Warsaw University of Technology, Nowowiejska Str. 15/19, 00-665 Warsaw, Poland; 2 School of Computer Science & Technologies, VIZJA University, Okopowa Str. 59, 01-043 Warsaw, Poland; j.niepostyn@vizja.pl

**Keywords:** UML class diagrams, software architecture metrics, information content, normalized entropy, software measurement

## Abstract

Entropy-based metrics can provide a quantitative representation of the information content of UML models, but their practical usefulness depends on a systematic and reproducible calibration of the parameters used to construct the underlying probability distribution. This paper presents FBS26, an extension of the FBS metric that introduces calibrated weights for UML element kinds while retaining the normalized Shannon entropy formulation. The proposed calibration procedure combines three criteria: avoiding false lack-of-information cases, reducing non-monotonic behavior of the metric, and achieving a pronounced decrease in the metric for balanced diagram series. The criteria are applied according to a predefined lexicographic priority. The approach is evaluated using a controlled series of UML class diagrams and 123 UML class diagrams from four industrial software projects, selected from a repository containing more than 2000 UML diagrams from 18 projects. Architectural maturity labels, assigned independently by an experienced software architect, serve as reference data for examining empirical thresholds for structural information. The results show that systematic weight calibration removes the undesirable behavior observed for the considered FBS16 configurations and provides a more stable basis for comparing UML class diagrams. The industrial analysis further indicates differences in the structural entropy ranges of commonly distinguished diagram categories. FBS26, therefore, provides a quantitative information-content measure that can support expert-based assessment of the architectural maturity of UML class diagrams.

## 1. Introduction

Software architects routinely use UML class diagrams to describe software structure during early development stages [1,2,3]. In large projects, dozens or even hundreds of such diagrams may coexist, representing different viewpoints and levels of abstraction. Evaluating whether a diagram is sufficiently informative, complete, or mature is therefore an important task, yet in practice it is often performed subjectively and relies heavily on architects’ experience. Despite the increasing adoption of code-centric and AI-assisted development approaches, model-based engineering and UML remain widely used in safety-critical, enterprise, and government software projects, where architecture documentation and traceability are required [1,4]. In this work, the architectural maturity of a diagram is defined as the extent to which a class diagram provides sufficient architectural information for its intended development stage.

Recent surveys indicate that software modeling continues to evolve as a central research area, with increasing attention to model quality, model analysis, automation, and industrial applications, underscoring the enduring importance of quantitative techniques for model assessment [4,5].

Numerous software metrics have been proposed to support model assessment. Most of them focus on size, complexity, or the number of modeling elements. According to ISO/IEC 25010 [6], maintainability and functional suitability are core pillars of software product quality. Recent surveys indicate that most software model quality metrics still focus on structural complexity, maintainability, and size characteristics [7,8], while quantitative assessment of information content remains relatively underexplored [9,10]. While these measures are useful for estimating effort, maintainability, or structural complexity, they offer limited insight into the amount of information a diagram conveys. As a result, two diagrams of similar size may differ substantially in their informational value. Consequently, existing metrics offer limited support for assessing architectural maturity, as diagram completeness cannot be inferred from size or structural complexity alone.

Research Gap. Information-theoretic approaches offer a promising alternative because they assess the distribution and concentration of modeling elements rather than merely counting them. Several entropy-based UML metrics have been reported in the literature, demonstrating that entropy can be successfully applied to software models [10,11]. Nevertheless, important challenges remain. In particular, the influence of element kinds on the entire UML diagram is rarely considered, making the resulting measurements difficult to reproduce and compare across projects. Furthermore, relatively little attention has been paid to understanding how weighting schemes influence metric behavior, while empirical validation is frequently limited to relatively small datasets.

To investigate the information content of UML models, the authors previously proposed FBS16 [12], an entropy-based measure capable of distinguishing diagrams that contain meaningful information from those that lack it. Although the initial results were encouraging, subsequent experiments revealed several practical limitations. The metric proved highly sensitive to manually assigned weights, and some weight configurations produced undesirable effects, including false lack-of-information cases and non-monotonic changes in metric values.

This paper presents FBS26, an extension of the previous FBS16 metric that introduces a systematic and reproducible procedure for selecting UML element-kind weights. The goal is not to redefine the entropy measure itself, but to reduce undesirable metric behavior resulting from manually selected weights and to make the resulting measurements more consistent and interpretable. The calibration procedure is deterministic once the candidate weight space, benchmark diagrams, and priority criteria have been specified.

The resulting information-content measure can subsequently be used as quantitative evidence in the assessment of architectural maturity. In this study, maturity is not defined by the entropy value alone; instead, FBS26 values are compared with expert-based reference classifications and used to investigate empirical maturity thresholds for different categories of UML class diagrams.

The study addresses the following research questions:

1. Can a systematic weight-selection procedure reduce false lack-of-information cases observed in entropy-based UML metrics?
2. Can the proposed procedure reduce the non-monotonic behavior of metric values for diagrams of comparable concentration?
3. Do FBS26 structural entropy values differ systematically between UML class diagrams assigned different architectural-maturity labels in the evaluated industrial dataset?
4. Do commonly distinguished UML class-diagram categories exhibit different empirical ranges of FBS26 structural entropy?

For empirical evaluation, we use a repository containing more than 2000 UML diagrams originating from 18 software projects [13]. The repository was not used in its entirety to validate FBS26, since a typical project contains only a small number of class diagrams. Instead, four projects containing substantially larger numbers of UML class diagrams were selected to provide a sufficiently diverse set of class-diagram instances for empirical analysis. This purposive selection favors projects with a sufficiently large number of class diagrams and, therefore, should not be interpreted as a statistically representative sample of the complete repository. The projects contain 48 (ECS), 28 (EIRZ), 11 (NIK), and 36 (SWPL) class diagrams, respectively, for a total of 123 UML class diagrams in the validation set. This selection provides a substantially richer basis for evaluating the behavior of FBS26 than would be obtained from projects containing only a few class diagrams, while retaining the projects’ original modeling context.

Thus, the size of the repository should not be interpreted as the size of the validation sample: the repository provides the source population, whereas the 123 selected class diagrams constitute the empirical validation dataset used in this study.

The main contributions of this paper are:

- a systematic and reproducible procedure for calibrating UML element-kind weights,
- an extension of the FBS entropy-based metric with calibrated weights for UML class diagrams,
- validation on a significant sample of diagrams from industrial projects.

We propose FBS26 as an information-content measure for UML models, extending the FBS approach through an empirically selected weighting scheme for UML modeling elements. Unlike conventional size and complexity metrics, FBS26 is intended to quantify the information content represented by a model rather than merely the number or structural complexity of its elements.

We provide reference threshold values for FBS26 for different types of UML class diagrams. These thresholds are based on the authors’ practical experience with UML modeling and were subsequently confirmed through their application to the real-world projects considered in this study. The thresholds are therefore intended as empirically grounded reference values rather than as the outcome of a formally defined threshold-estimation procedure.

The remainder of this paper is organized as follows. Section 2 presents related work, with particular emphasis on entropy-based software, UML model quality metrics, complexity measures, and empirical approaches to UML model evaluation. Section 3 introduces the FBS26 metric and describes the proposed weight-calibration procedure, including the criteria for selecting the element weights. Section 4 presents the empirical evaluation, including the construction of the evaluation dataset, the expert-based maturity assessment, and the analysis of FBS26 results for UML class diagrams from industrial software projects. Section 5 discusses the results and their implications, including the interpretation of architectural maturity thresholds and the limitations of the proposed approach. Finally, the paper concludes and outlines directions for future research.

## 2. Related Work

Software modeling remains an active research area, with increasing attention to model quality, model analysis, automation, and empirical evaluation [14,15,16,17]. Numerous metrics have been proposed to support the quantitative assessment of UML models. Although these metrics differ in their objectives and mathematical foundations, they can be broadly classified into three categories based on the properties they evaluate: size-based, complexity-based, and entropy-based.

### 2.1. Size-Based Metrics

Early approaches to UML measurement focused primarily on quantifying the size of software models. These measures typically count modeling elements, such as classes, attributes, operations, associations, and inheritance relationships, and use these counts as indicators of model size, development effort, or documentation characteristics [18,19,20]. Representative approaches include metrics proposed for UML by Marchesi and Genero, as well as object-oriented metrics derived from the CK metric suite and related to UML models.

Size-based metrics are straightforward to compute and remain useful for characterizing model growth and estimating development effort. However, they provide only limited information about the actual information content of a diagram. Two models containing similar numbers of UML elements may represent substantially different amounts of architectural information. Consequently, element counts alone provide limited support for assessing whether a diagram contains sufficient information for its intended development stage.

### 2.2. Complexity Metrics

A second major group of approaches evaluates the structural complexity of UML models. These metrics consider properties such as coupling, cohesion, inheritance, abstraction, and maintainability rather than simply counting modeling elements.

Coupling metrics, such as CBO from the CK metric suite [20] and DCC from QMOOD [21], quantify dependencies between classes and are commonly used as indicators of design complexity and maintainability. Similarly, cohesion metrics such as LCOM [20] and CAM [21] characterize relationships among elements within classes and complement coupling measures in assessing software quality. Inheritance metrics, including DIT and NOC [20] and MFA [21], characterize inheritance hierarchies and their influence on object-oriented design.

QMOOD [21] is an influential quality-oriented approach that maps coupling, cohesion, inheritance, abstraction, polymorphism, and encapsulation to higher-level quality attributes, including reusability, flexibility, understandability, functionality, extendibility, and effectiveness. Other studies have further investigated quantitative relationships between object-oriented metrics and software quality attributes [19,20,21].

Although complexity metrics characterize important structural properties of UML models, they do not directly measure the information content represented by a diagram [22]. Complexity and information content should therefore not be treated as equivalent properties. A diagram may exhibit relatively high structural complexity without necessarily containing a proportionally high amount of architectural information.

### 2.3. Entropy-Based Metrics

Information-theoretic approaches provide a different perspective by evaluating the distribution and diversity of modeling elements rather than their absolute numbers. Following Shannon’s formulation of information entropy [23,24] and Harrison’s early application to software complexity [25], entropy-based measures have subsequently been investigated in software engineering and UML-related contexts [10,11,26,27].

Entropy-based measures are particularly relevant to UML models because they can distinguish between diagrams having similar numbers of elements but different distributions of element kinds. In this sense, entropy provides information that cannot be obtained from simple element counts alone. Yi et al. investigated the use of metrics for UML class diagrams [11], while subsequent work applied information entropy to multi-attribute decision making for UML class diagram metrics [26]. These studies demonstrate the potential of information-theoretic approaches for quantitative analysis of UML models.

Entropy has also been applied at the software architecture level. For example, Niepostyn and Daszczuk investigated entropy as a measure of consistency in software architecture [10]. More generally, information-theoretic measures have been investigated for software measurement and source-code analysis, demonstrating that entropy remains an active research topic in software engineering.

However, entropy-based measurements require a definition of the probability distribution from which entropy is calculated. In UML applications, this distribution may depend on how different modeling elements are treated and their relative importance. Consequently, assumptions concerning element weights can influence the resulting metric values. This issue is particularly important when the metric is intended to compare diagrams with different compositions or abstraction levels.

Recent work has also emphasized the importance of empirically validating information-theoretic software measures. Torres et al. investigated information-theoretic measures in software engineering and highlighted the limited extent of rigorous empirical validation in this research area [28]. This observation is particularly relevant when entropy-based metrics are intended to support practical software engineering decisions.

### 2.4. Recent Advances in UML and Model Quality Metrics

Recent work has also addressed broader approaches for model quality assessment, analysis, automation, and empirical validation [15,17,28]. Current work addresses not only individual metric definitions but also model consistency, architecture evolution, model analytics, and the integration of quantitative assessment into software engineering processes.

Model-driven engineering remains an active research area. The research demonstrates the role of UML and other modeling approaches in architecture-centric development and software engineering, while research increasingly investigates automated model analysis and the interaction between modeling and emerging software engineering technologies [15,29]. These developments indicate that quantitative model assessment is increasingly considered part of broader model-driven engineering workflows rather than an isolated measurement activity.

Another noticeable trend is the growing emphasis on empirical validation. Systematic reviews of UML model quality research have identified a need for stronger empirical evidence to support claims about model quality and quality metrics [16]. More recent empirical research has likewise emphasized the importance of systematic evaluation and suitable datasets when investigating model-related approaches. Thus, the practical applicability of software metrics should be supported by empirical evidence rather than relying exclusively on theoretically constructed examples.

Modern model-driven approaches increasingly combine architectural models with quantitative metrics to evaluate predefined quality attributes and support engineering decisions throughout the software lifecycle [15,28]. Such approaches demonstrate the continuing relevance of quantitative architectural assessment.

Nevertheless, these approaches primarily address predefined quality attributes, architectural properties, or structural characteristics. Comparatively little attention has been devoted to explicitly quantifying the information content of UML class diagrams and relating such measurements to architectural maturity [11,26,30].

The authors previously addressed this problem by proposing FBS16, an entropy-based metric for assessing the information content of UML class diagrams [12]. FBS16 demonstrated the applicability of normalized entropy to UML diagrams. However, subsequent experiments revealed that the metric’s behavior was sensitive to the manually assigned weights for UML elements. These observations motivated the development of FBS26, which retains the information-theoretic foundation of FBS16 while introducing a systematic procedure for calibrating the weights assigned to UML element kinds.

### 2.5. Existing Approaches and Research Gap

The reviewed approaches can be summarized into three principal categories, as shown in Table 1.

The comparison highlights that these metric categories address complementary aspects of UML models. Size-based metrics quantify the scale of a model, while complexity-based metrics characterize structural relationships and properties. Entropy-based metrics provide an information-theoretic perspective by considering the distribution of modeling elements.

However, comparatively little attention has been devoted to explicitly quantifying the information content of UML class diagrams and relating such measurements to architectural maturity. Moreover, the choice of weights assigned to different UML element kinds may influence entropy-based measurements and therefore requires systematic treatment. The literature also indicates a need for stronger empirical validation of software metrics, particularly when they are intended for practical application [16].

FBS26 addresses these gaps by combining normalized entropy with a systematic procedure for calibrating UML element weights. Unlike size-based metrics, the proposed approach does not rely solely on the number of modeling elements. Unlike conventional complexity metrics, it is not intended primarily to measure coupling, cohesion, inheritance, or maintainability. Instead, FBS26 focuses on the information content represented by a UML class diagram and uses this information as a quantitative basis for assessing architectural maturity.

The empirical evaluation follows the methodological approach identified in recent research, combining controlled experiments with analysis of a large industrial dataset. In particular, FBS26 is evaluated using artificially constructed diagram series as well as industrial diagrams selected from a repository of 18 software projects. This combination enables examination of both the metric’s controlled behavior and its applicability to diagrams observed in industrial software development.

## 3. Methodology

### 3.1. Proposed Framework

Within the proposed framework, architectural maturity is investigated using the information content represented by a UML class diagram. FBS26 provides a quantitative measure of this information content, while architectural maturity is interpreted using reference assessments and empirical thresholds established for the analyzed diagram categories.

The FBS26 metric is an extension of the previously proposed FBS16 [12]. Both metrics are based on the information-theoretic interpretation of UML class diagrams and use normalized Shannon entropy to characterize the distribution of UML element kinds across three dimensions: functionality, behavior, and structure. The entropy calculation itself remains unchanged between the two metric versions. The essential difference concerns the method used to select the element kind weights to obtain the metric’s expected output characteristics.

The proposed FBS26 extends the previously developed FBS16 by introducing a systematic and reproducible procedure for calibrating UML element weights. While the underlying entropy formulation remains unchanged, the proposed approach modifies the way probabilities are computed by replacing manually selected weights with values obtained through a systematic calibration procedure.

**Figure 1:**
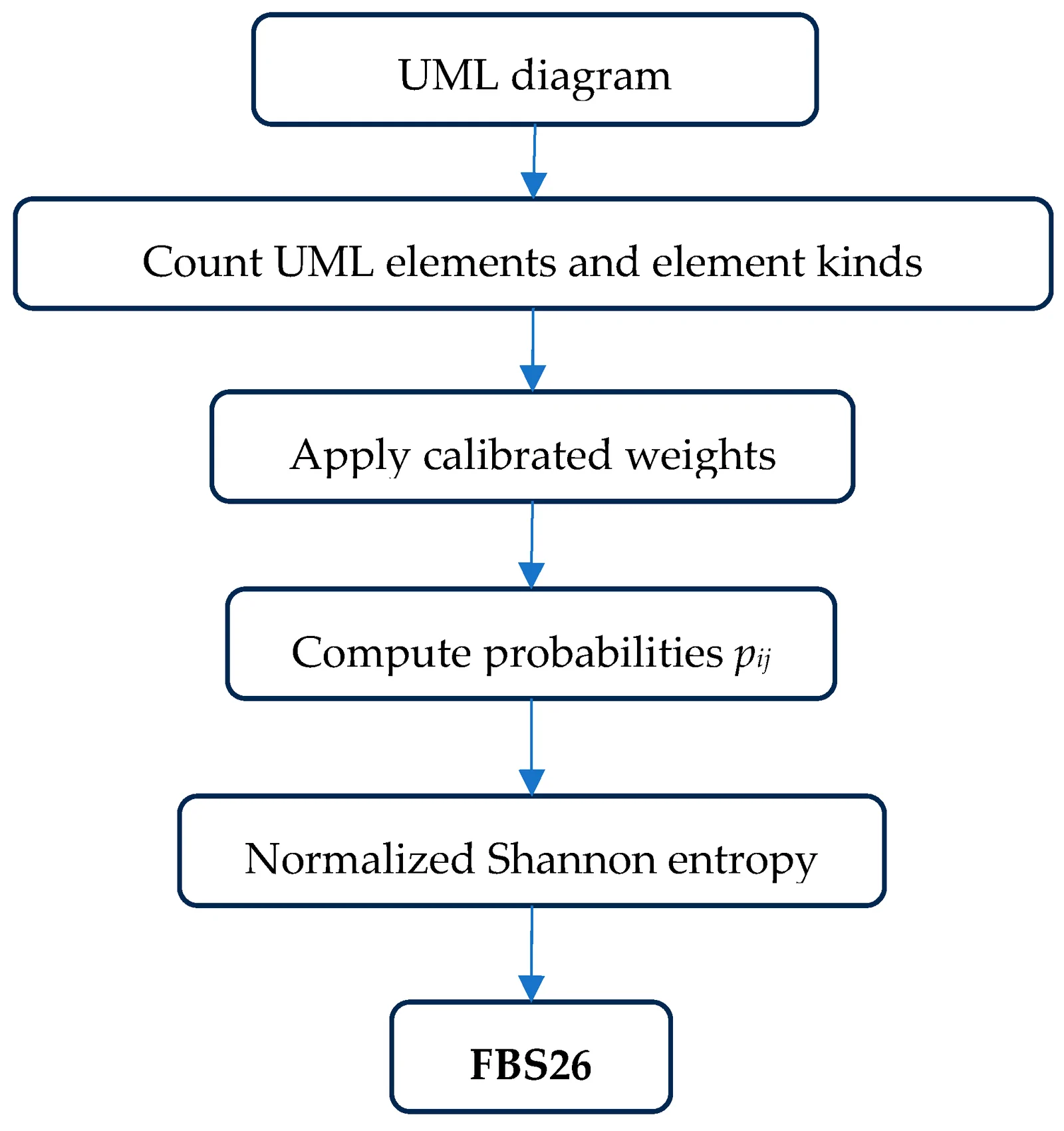
Workflow of FBS26.

The overall workflow of FBS26 is illustrated in Figure 1. The process begins by identifying the occurrence of each UML element kind within a class diagram. Each occurrence is multiplied by its calibrated weight, producing weighted frequencies that are subsequently normalized to obtain the probability distribution used in entropy calculation. Finally, normalized Shannon entropy is computed to quantify the information content of the analyzed diagram.

The original entropy formulation derives the probability distribution directly from the occurrence counts of UML element kinds, without incorporating element weights. In FBS26, entropy is computed over the individual UML elements of a diagram, while the relative importance of each element is determined by the weight assigned to its UML element kind.

Let *m* denote the total number of UML elements in a class diagram and *n* the number of distinct UML element kinds considered by the metric. Let *i* = 1, …, *m* index individual UML elements and *k* = 1, …, *n* index UML element kinds. Let *t*(*i*) denote the UML element kind to which the individual element *i* belongs. Let *c_k_* denote the number of occurrences of UML elements of kind *k*, such that

(1) $$\sum_{k=1}^{n}c_{k}=m.$$

Let *w_kj_* denote the weight assigned to UML element kind *k* when evaluating FBS dimension *j*, and let *q_kj_* denote the dimension coefficient indicating whether element kind *k* contributes to dimension *j*.

The main modification introduced in FBS26 concerns the probability distribution rather than the Shannon entropy function itself. The motivation for this modification is that the occurrence frequency of a UML element kind alone does not account for differences in the relative importance of UML element kinds for a given FBS dimension. FBS26, therefore, combines the occurrence count *c_k_* with the corresponding element-kind weight *w_kj_*. Thus, the weighted frequency of element kind *k* for dimension *j* is expressed by the product

*c_k_w_kj_*. (2)

For an individual UML element *i*, belonging to element kind *t*(*i*), its contribution to dimension *j* is determined by the corresponding element-kind weight and dimension coefficient. The decision-matrix element is therefore defined as

(3) $$a_{\mathrm{ij}}^{26}=w_{t\left(i\right)j}q_{t\left(i\right)j}.$$

Thus, individual elements belonging to the same UML element kind receive the same weight and dimension coefficient, while each occurrence contributes separately to the entropy calculation.

The decision matrix is normalized separately for each FBS dimension. First,

(4) $$M_{j}^{26}=\underset{1\leq i\leq m}{\max}\left(a_{\mathrm{ij}}^{26}\right),$$

and

(5) $$r_{\mathrm{ij}}^{26}=\frac{a_{\mathrm{ij}}^{26}}{M_{j}^{26}}.$$

The probability distribution is then obtained by column normalization:

(6) $$p_{\mathrm{ij}}^{26}=\frac{r_{\mathrm{ij}}^{26}}{\sum_{l=1}^{m}r_{lj}^{26}}.$$

After canceling the common normalization factor $M_{j}^{26}$, this gives

(7) $$p_{\mathrm{ij}}^{26}=\frac{w_{t\left(i\right)j}q_{t\left(i\right)j}}{\sum_{l=1}^{m}w_{t\left(l\right)j}q_{t\left(l\right)j}}.$$

Because each UML element kind *k* occurs *c_k_* times, the equation can equivalently be expressed in terms of UML element kinds and their occurrence counts:

(8) $$p_{\mathrm{kj}}^{26}=\frac{c_{k}w_{\mathrm{kj}}q_{\mathrm{kj}}}{\sum_{l=1}^{n}c_{l}w_{lj}q_{lj}},k=1,\ldots ,n.$$

This formulation explicitly distinguishes the two levels used by FBS26. The probability distribution is defined over the *n* UML element kinds, whereas the weights are assigned to the individual kinds. Thus, the occurrence frequency and the calibrated importance of each UML element kind jointly determine its contribution to the entropy.

Importantly, the weighting mechanism does not modify the Shannon entropy function. FBS26 uses the same normalized entropy formulation:

(9) $$E_{j}^{26}=-\frac{1}{\ln\left(m\right)}\sum_{k=1}^{n}p_{\mathrm{kj}}^{26}\ln\left(p_{\mathrm{kj}}^{26}\right).$$

The normalization factor ln(*m*) corresponds to the number of individual UML elements, but the entropy is calculated over element kinds (1, …, *n*). As usual in Shannon entropy, terms with $p_{\mathrm{ij}}^{26}=0$ are defined to contribute zero, i.e., 0∙ln0 = 0. Substituting the weighted probability distribution gives

(10) $$E_{j}^{26}=-\frac{1}{\ln\left(m\right)}\sum_{k=1}^{n}\left(\frac{c_{k}w_{\mathrm{kj}}q_{\mathrm{kj}}}{\sum_{l=1}^{n}c_{l}w_{lj}q_{lj}}\right)\ln\left(\frac{c_{k}w_{\mathrm{kj}}q_{\mathrm{kj}}}{\sum_{l=1}^{n}c_{l}w_{lj}q_{lj}}\right).$$

This formulation constitutes the principal mathematical modification introduced by FBS26: the entropy is calculated over individual UML elements, while the probability assigned to each element incorporates the weight associated with its UML element kind. The Shannon entropy function itself remains unchanged. The final FBS26 metric is consequently defined as

(11) $$FBS26=(E_{F}^{26},E_{B}^{26},E_{S}^{26}).$$

In FBS16, the weighting schemes were selected with the primary objective of avoiding false lack-of-information cases. FBS26 extends this objective by considering additional properties of metric behavior, including monotonicity and the rate at which the metric decreases as the amount of represented information increases. The procedure used to determine the weights (*w_t_*_(*i*)*j*_) is described in the following section.

### 3.2. Weight Selection in FBS16 and FBS26

The element weights introduced in FBS26 are parameters used to construct the weighted element frequencies and, consequently, the probability distribution supplied to the entropy function. They are not additional parameters of the Shannon entropy itself. Their values are determined through a calibration procedure based on the observed behavior of the metric.

Let (*W*) denote the set of candidate configurations of element kind weights (*w_kj_*). For each configuration (*W*), the corresponding FBS metric values are calculated for the considered series of UML class diagrams. The quality of a particular configuration is then evaluated using criteria describing undesirable metric behavior.

In the original FBS16 metric, the primary criterion for selecting the element weights was to avoid false lack-of-information cases. Let *FLI*(*W*) denote the number of false lack-of-information cases obtained for a candidate weight configuration (*W*). The FBS16 calibration can therefore be expressed as the following optimization problem:

*W*^16^ = *arg min_W_FLI*(*W*). (12)

Thus, the selected configuration is the one that minimizes the number of cases in which a diagram containing relevant information is incorrectly assigned an entropy value indicating a lack of information.

FBS26 extends this procedure by considering not only false lack-of-information cases but also the behavior of metric values across diagram series. In particular, adding or increasing the amount of represented information should not produce undesirable increases in the metric value. Consequently, the calibration procedure introduces additional criteria that describe the metric’s monotonic behavior.

Let *ANMV*(*W*) (*Average Number of Monotonicity Violations*) denote the measure of non-monotonic metric behavior for a candidate configuration (*W*). *ANMV* is defined as:

(13) $$\mathrm{ANMV}\left(W\right)=\frac{1}{|S|}\sum_{s\in S}V_{s}\left(W\right),$$

where

(14) $$V_{s}\left(W\right)=\sum_{r}1\left[{FBS}_{W}\left(D_{r+1}\right)>{FBS}_{W}\left(D_{r}\right)\right].$$

Thus, *ANMV* measures the average number of monotonicity violations per calibration series. A lower value is preferred, with (*ANMV*(*W*) = 0) indicating that no monotonicity violation was observed in the considered artificial series.

The calibration objective can then be expressed as a multi-criterion optimization problem:

*W*^26^ = *arg min_W_*[*FLI*(*W*), *ANMV*(*W*)] (15)

where the first criterion preserves the original requirement of FBS16, while the second criterion evaluates the consistency of metric behavior when the amount of information represented in a diagram increases.

When evaluating balanced diagram series, an additional criterion can be used to distinguish configurations that yield a stronger, more consistent decrease in the metric. Let *DMBD*(*W*) (*Decrease in Metric for Balanced Diagrams*) denote the corresponding criterion, referred to as the maximum decrease in monotonicity for balanced diagrams. Balanced diagrams belong to a *Diag* series—see later. The complete calibration procedure can consequently be represented as

*W*^26^ = *arg min_W_*[*FLI*(*W*), *ANMV*(*W*), −*DMBD*(*W*)]. (16)

The three criteria represent complementary requirements. *FLI* evaluates whether the metric correctly identifies the presence of information, *ANMV* evaluates whether the metric behaves monotonically when diagrams are enriched with additional information, and *DMBD* evaluates the magnitude and consistency of the metric decrease for balanced diagram series.

This formulation also clarifies the methodological difference between FBS16 and FBS26. The entropy function is identical in both metrics, and the weighted element kind frequencies (*c_k_w_kj_*) and dimension coefficients (*q_t_*_(*i*)*j*_) retain the same meaning.

Thus, FBS26 can be understood as an extension of FBS16 at the level of weight calibration, rather than as a modification of the underlying entropy measure.

The detailed definition of the monotonicity criteria used for weight calibration is presented in Section 3.3. In particular, the *DMBD* criterion is introduced there to evaluate the decrease in metric values for balanced diagram series and to distinguish weight configurations that provide a stronger and larger observed decrease response to increasing diagram size. This separation allows the mathematical definition of FBS26 to be distinguished from the empirical procedure used to select its element weights.

### 3.3. Decrease in Metric for Balanced Diagrams

The second aspect of the calibration concerns the rate at which the metric decreases when the number of UML elements increases. This property is particularly important for balanced diagram series, in which the proportions of the considered UML element kinds remain approximately constant while the overall number of elements increases. Such series make it possible to examine the response of the metric to an increase in diagram size without introducing substantial changes in the relative distribution of element kinds.

Let (*D*_1_, *D*_2_, …, *D_s_*) denote a balanced series of UML class diagrams ordered by increasing number of elements (we use only odd values, so (*D*_1_, *D*_3_, …, *D_s_*), where *s* is odd). For a given candidate weight configuration (*W*), let

*FBS_W_*(*D_r_*) (17)

denote the value of the considered FBS component for diagram (*D_r_*). Since the metric is intended to decrease as the amount of represented information increases, the difference between two consecutive diagrams is defined as

Δ*_W_*_(*r*)_ = *FBS_W_*(*D_r_*) − *FBS_W_*(*D_r_*_+2_) (18)

for (*r* = 1, 3, …, *s* − 2).

A positive value of (Δ*_W_*_(*r*)_) indicates the expected decrease in the metric, whereas a negative value indicates a non-monotonic transition. The magnitude of the positive difference represents the rate at which the metric decreases between two consecutive balanced diagrams.

The *DMBD* criterion is used to characterize the strongest decrease observed in a balanced diagram series. It can therefore be expressed as

*DMBD*(*W*) = *max_r_*Δ*_W_*_(*r*)_ (19)

where the maximum is calculated over all consecutive pairs of diagrams in the considered balanced series.

Because the artificial diagram series is constructed with an odd number of complexity levels, the central diagram serves as the most important reference point of the series. In particular, for the five-level series considered in the calibration procedure, the third diagram (*D*_3_) represents the central level and is therefore given greater importance than the extreme cases. The behavior around the central diagram, especially the changes leading to and following *D*_3_, provides a more representative indication of the metric’s response to increasing structural complexity. If Δ*_W_*_(3)_ is comparable for two sets of *W*, Δ*_W_*_(5)_ can be considered, and so on. Because there are four series (*Diag*, *Op*, *AssAt*, *ClassAss*), the average value is applied.

The criterion provides an additional way to distinguish candidate weight configurations that may satisfy the basic requirement of monotonicity yet differ substantially in the metric’s sensitivity to increases in diagram size. A configuration that produces only very small decreases may technically satisfy the monotonicity requirement while providing limited discrimination between diagrams of different sizes. Conversely, a configuration producing a clear decrease across the balanced series provides a stronger response to increasing amounts of represented information.

*DMBD* is therefore considered together with the false lack-of-information and monotonicity criteria during weight calibration. The purpose of this criterion is not to modify the entropy function, but to select among candidate element-weight sets those for which the resulting metric exhibits an appropriate decrease when the number of UML elements increases while their relative proportions remain balanced.

The calibration of the UML element weights involves three objectives: minimizing the false-lack-of-information rate (*FLI*), minimizing the number of non-monotony cases (*ANMV*), and maximizing the distance between the metric values obtained for balanced diagrams having different numbers of elements (*DMBD*). These objectives are not fully aligned, and an exhaustive search for a simultaneous optimum would therefore constitute a multi-objective optimization problem. In practice, a single weight configuration may improve one criterion while degrading another, making the selection of a single optimum across the three criteria non-trivial.

To obtain a deterministic and reproducible selection rule, we therefore impose an explicit hierarchy on the three objectives. The false-lack-of-information *FLI* rate is treated as the primary criterion, the average number of monotonicity violations *ANMV* as the secondary criterion, and the distance between metric values for different maturity levels as the tertiary criterion. This hierarchy is implemented through the following scalar objective function:

*O* = lexmin*_W_*(*FLI*(*W*), *ANMV*(*W*), −*DMBD*(*W*)) (20)

The negative sign before *DMBD* reflects the fact that the function of entropy decreases in the number of elements (except non-monotonic cases). Thus, the objective function does not attempt to assign an arbitrary trade-off between the three criteria; instead, it provides the predefined priority order.

### 3.4. Weight Calibration

Preliminary experiments performed during the development of FBS16 demonstrated that manually assigned UML element weights significantly influence metric behavior. Certain weight configurations produced two undesirable effects. First, diagrams containing meaningful information occasionally obtained entropy values indicating a lack of information. Second, some diagram transformations resulted in non-monotonic changes in entropy, with the addition of meaningful information unexpectedly increasing the entropy value.

To eliminate these effects, a systematic calibration procedure was developed.

Instead of relying solely on expert judgment, calibration was performed using a collection of artificial UML class diagrams designed to expose problematic situations observed in previous experiments. These reference diagrams are presented in Table 2.

The values in Table 2 are calculated from the probability distribution over UML elements. The entropy reflects both the relative frequencies of the element kinds and their calibrated weights. For equal weights, a balanced distribution of the considered element kinds produces a high normalized entropy, whereas increasing concentration in one element kind reduces the entropy. This behavior explains the decrease in the *Diag* values as the diagram series becomes increasingly concentrated in the corresponding element kinds.

The calibration dataset consists of four complementary diagram families.

- *ClAss*—CLDs consisting of an increasing number of Classes and Associations,
- *AssAt*—CLDs consisting of 1 Class and an increasing number of Attributes and Associations,
- *Op*—CLDs consisting of 1 Class and an increasing number of Operations,
- *Diag*—CLDs composed of an increasing, balanced number of Classes, Attributes, Operations, and Associations.

Together, these diagrams represent controlled model evolution scenarios in which the expected behavior of the metric is known a priori.

Several candidate weighting schemes were evaluated using these reference diagrams. The objective was to identify weights satisfying three conditions:

- minimizing false lack-of-information cases (red in Table 2),
- reducing non-monotonic entropy behavior (absent in the presented weight sets in Table 2),
- preserving intuitive relationships between model evolution and information content (decreasing fastest in element count—grey background in Table 2).

Eight representative weight sets (F,B,S) were selected from a larger pool. Here, Association refers to a relationship that connects Classes and affects only the structural dimension; other UML relationship details are ignored for simplicity. The possible weights for each CLD element were: Class {(0,0,1)…(0,0,5)}, Attribute {(0,0,1)…(0,0,3)}, Association {(0,0,1)…(0,0,7)}, and Operation {(1,1,1)…(1,1,3)}, with the larger maximum for Association reflecting its link between two elements.

The eight selected sets are given in Table 3.

The resulting weighting scheme was adopted throughout the remainder of the study and subsequently evaluated using both artificial benchmark diagrams and industrial UML repositories.

**Figure 2:**
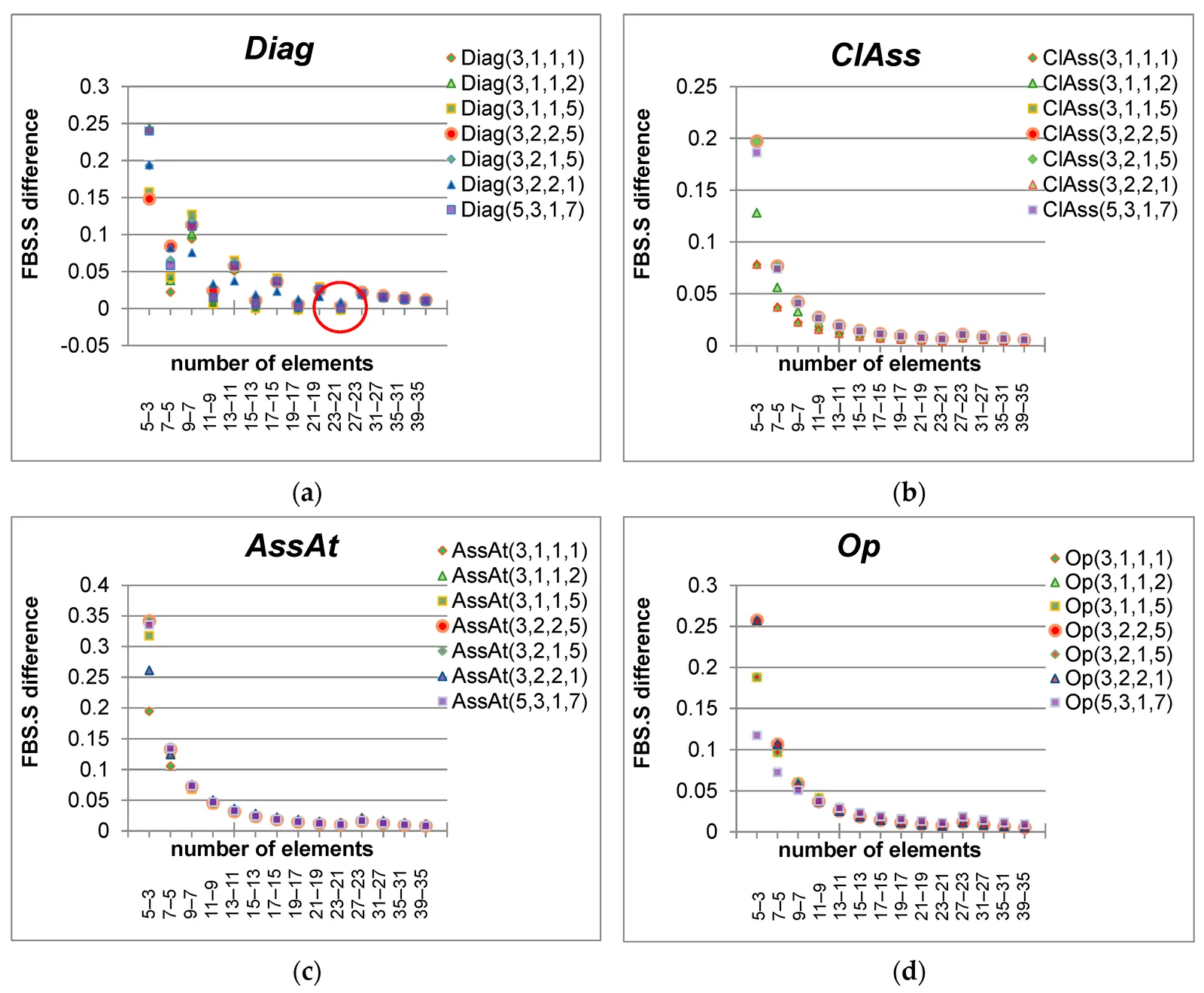
The differences between neighboring CLDs’ FBS.S metric values depending on the number of elements for CLDs. The element weights are given in parentheses. (**a**) All *Diag* CLDs except *Diag*(1,1,1,1), (**b**) *ClAss* diagrams, (**c**) *AssAt* diagrams, (**d**) *Op* diagrams. The labels in the graph legends are the series names, followed by the weight sets in parentheses. Non-monotonic *DMBD* between samples 23 and 21 in *Diag* series is surrounded by a red circle.

The eight selected weight sets were further analyzed for their behavior as a function of the number of elements. The best set should decrease fastest in the number of elements. Figure 2 presents the differences (Δ*_W_*_(*r*)_) between consecutive diagrams in each artificial series. A positive value indicates the expected decrease in the FBS.S value when the amount of represented information increases, whereas a negative value indicates a monotonicity violation. Values close to zero indicate only a weak numerical decrease and therefore provide little separation between consecutive diagrams. Larger positive values indicate a stronger decrease and contribute positively to the *DMBD* criterion.

Each curve corresponds to one candidate weight set, identified by the four structural weights shown in parentheses. The figure, therefore, provides a visual representation of the behavior quantified numerically by the calibration criteria. Weight sets exhibiting negative values are excluded by the monotonicity requirement, while sets with very small positive differences provide weaker discrimination between consecutive balanced diagrams.

For the *ClAss* series, the weight set (3,2,2,5) provides the most consistent performance overall, leading to its selection for FBS26 calculations. The comparison of the eight sets under evaluation is given in Table 4.

The proposed automation concerns the selection of UML element weights and, consequently, the objectification of the weight-calibration procedure. It does not eliminate the need for expert judgment when determining the threshold of architectural maturity. The maturity threshold should be established by an experienced software designer who can relate the metric values to the intended purpose and abstraction level of a diagram. In the present study, the element weights were automatically determined using the proposed calibration procedure. During this calibration process, several distinct types of UML class diagrams were identified for which different architectural-maturity thresholds appear to be appropriate (see Section 4.2). The FBS values obtained in all three dimensions—Functionality, Behavior, and Structure—can support the identification and differentiation of these diagram types. In particular, the FBS value in the Structural dimension can serve as an additional means of identifying diagram types by their entropy, provided the diagram represents an architecturally mature model. Thus, FBS26 does not prescribe a universal maturity threshold; rather, it provides quantitative information to help an expert establish thresholds appropriate for particular types of UML class diagrams.

## 4. Experimental Evaluation

### 4.1. Verification Using Artificial UML Diagrams

The first stage of the evaluation examined whether the proposed calibration procedure improves the metric’s internal behavior. The artificial diagram series introduced in Section 3.2 provides a controlled environment in which the expected entropy changes are known in advance.

The comparison of FBS26 with FBS16 demonstrates a substantial improvement over the previous metric. False lack-of-information cases were eliminated by selecting the correct weight sets. Non-monotonic entropy transitions were eliminated in the evaluated artificial diagram series, although such transitions may still occur for deliberately constructed pathological diagram configurations. More importantly, entropy values evolved consistently with intuitive expectations: diagrams enriched with additional UML elements generally yielded lower entropy values, indicating a higher concentration of information under the FBS26 formulation.

These results indicate that the systematic calibration procedure improves the stability and interpretability of the entropy-based measurement.

Although the artificial experiments provide controlled evidence of improved metric behavior, they cannot by themselves demonstrate practical applicability. For this reason, the second stage of the evaluation was conducted using industrial UML models.

### 4.2. Validation Using Industrial UML Projects

The empirical validation of FBS26 was conducted on a subset of a larger UML model repository of complex IT projects between 2005 and 2019, involving large systems with 5+ modules, built over 3+ years by teams of 30+ developers. The repository contains more than 2000 UML diagrams originating from 18 software projects. However, these diagrams include different UML diagram types, and a typical project contains only a limited number of class diagrams. Consequently, the entire repository was not treated as the validation sample for FBS26.

To obtain a sufficiently substantial and heterogeneous set of class diagrams, four projects containing comparatively large numbers of UML class diagrams were selected. The selected projects are ECS, EIRZ, NIK, and SWPL, containing 48, 28, 11, and 36 class diagrams, respectively. The resulting dataset comprises 123 UML class diagrams.

The purpose of this selection was not to claim that the metric was validated on all diagrams contained in the repository, but to obtain a sufficiently substantial set of class diagrams for which the proposed metric could be evaluated across different modeling contexts and diagram types. The repository, therefore, serves as the source population, whereas the 123 selected class diagrams constitute the actual empirical validation dataset.

The verification procedure consists of two complementary stages. First, a controlled series of diagrams is used to examine whether FBS26 exhibits the expected behavior as the number and composition of UML elements are systematically varied. This stage enables assessment of the metric under controlled conditions and identification of undesirable behavior independent of the variability found in industrial models. Second, the metric is evaluated on the 123 class diagrams extracted from the four selected software projects. This stage examines whether the behavior observed in the controlled experiments is also present in diagrams originating from real software development projects.

The selected diagrams are subsequently analyzed using the FBS26. The resulting values are used not only to assess the metric’s stability but also to investigate whether different types of UML class diagrams can be distinguished by their information distribution and architectural maturity. In particular, the analysis provides evidence for using different maturity thresholds for different types of class diagrams, rather than assuming a single universal threshold.

### 4.3. Expert-Based Maturity Assessment and Threshold Determination

The maturity thresholds presented in this study were not obtained through a formally defined threshold-estimation procedure. Instead, one of the authors assessed the maturity of all 123 class diagrams from the 18 projects considered in the study. Each diagram was classified as *mature* or *immature* based on its architectural characteristics, intended level of abstraction, and the author’s experience. These assessments were accepted in practice by the persons responsible for reviewing and approving the respective designs before implementation: mature diagrams were retained in the projects, whereas immature diagrams were rejected or redesigned.

After these assessments had been completed, the authors calculated FBS26.S for all 123 diagrams and examined its values in relation to the established maturity classifications. This analysis led to the proposal of reference thresholds for different types of class diagrams, selected to align with the previously established distinction between mature and immature diagrams. The comparison of FBS26.S values across diagram types also enables assessment of whether a common threshold is appropriate or whether diagrams serving different modeling purposes or representing different abstraction levels warrant separate reference values.

The resulting thresholds are therefore not claimed to be universal classification boundaries or statistically optimized values. They should be interpreted as empirically supported, dataset- and diagram-type-specific reference values reflecting the experience and project context represented in the study. They are intended as practical guidance for designers and reviewers rather than as requirements to be imposed on other projects. Other development teams may adopt these values, adapt them to their context, or establish their own thresholds based on their experience and project requirements.

For the article, we selected 55 diagrams from the 123 analyzed to illustrate our method. Those diagrams from 4 projects are presented in Table 5. For the architectural maturity analysis, the industrial CLDs were classified into six categories based on their architectural views: 5 conceptual, 24 business-view, 11 system-view, 6 ER, 6 BI, and 3 vocabulary diagrams. The maturity labels assigned by experienced architects were used as external reference labels to evaluate whether FBS26 distinguishes mature from immature diagrams.

**Figure 3:**
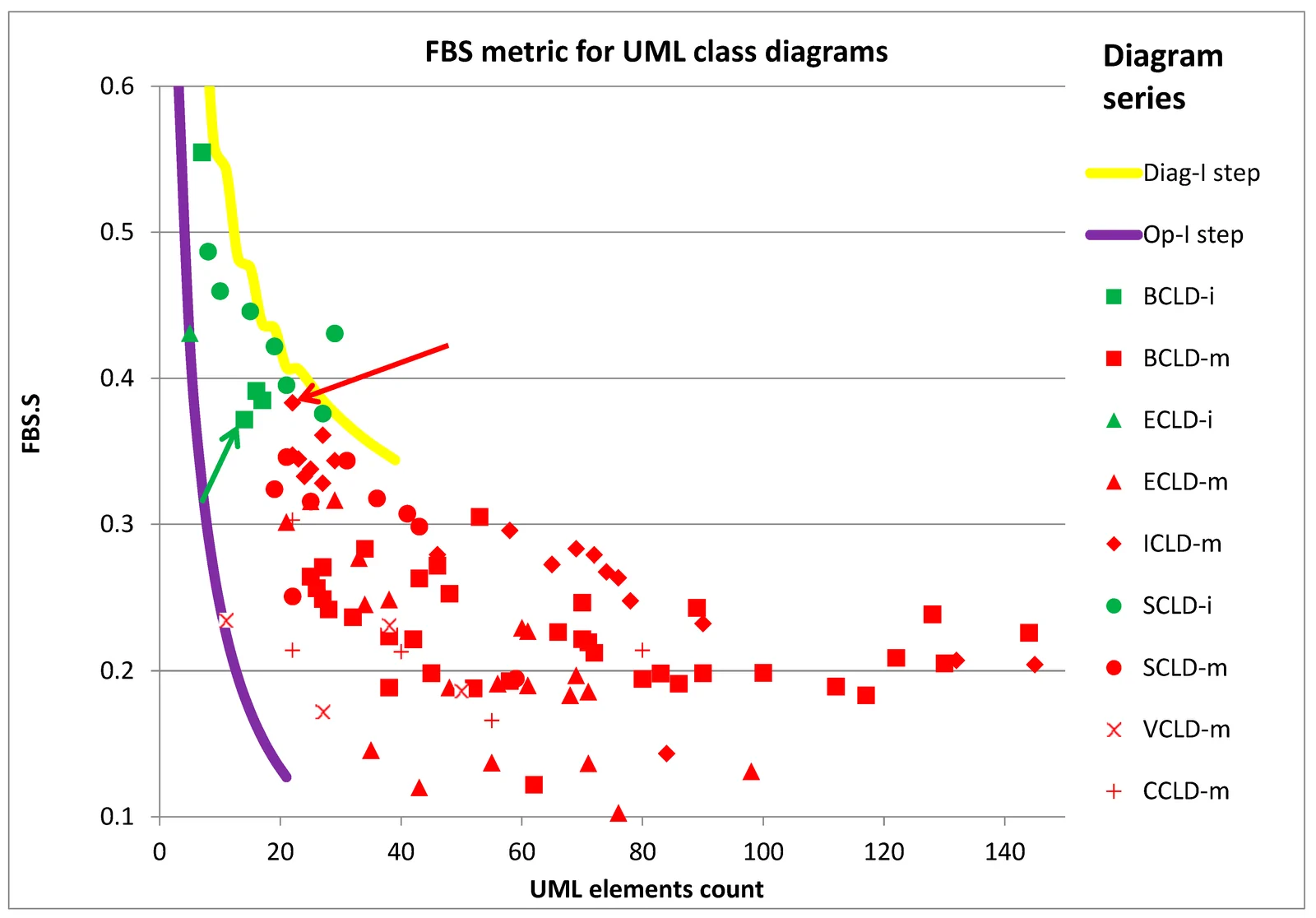
FBS26.S metric values of all 123 studied industrial CLDs. The last letter denotes: m—mature, i—immature. The meaning of the red and green arrows is described in the text.

Figure 3 visualizes FBS metric results for industrial CLDs. All FBS26 values fall between the *Diag* series (low concentration) and *Op* series (rapidly increasing concentration), showing that the constructed diagram series effectively approximate the range of values observed in the industrial diagrams. The acronyms denote: “BCLD”—general business class diagrams, “SCLD”—system class diagrams, “ICLD”—Business Intelligence tables (a type of business class diagrams), “ECLD”—Entity Relationships Class Diagrams (a type of business class diagrams), “VCLD”—vocabulary tables (a type of business class diagrams), and “CCLD”—conceptual class diagrams.

**Figure 4:**
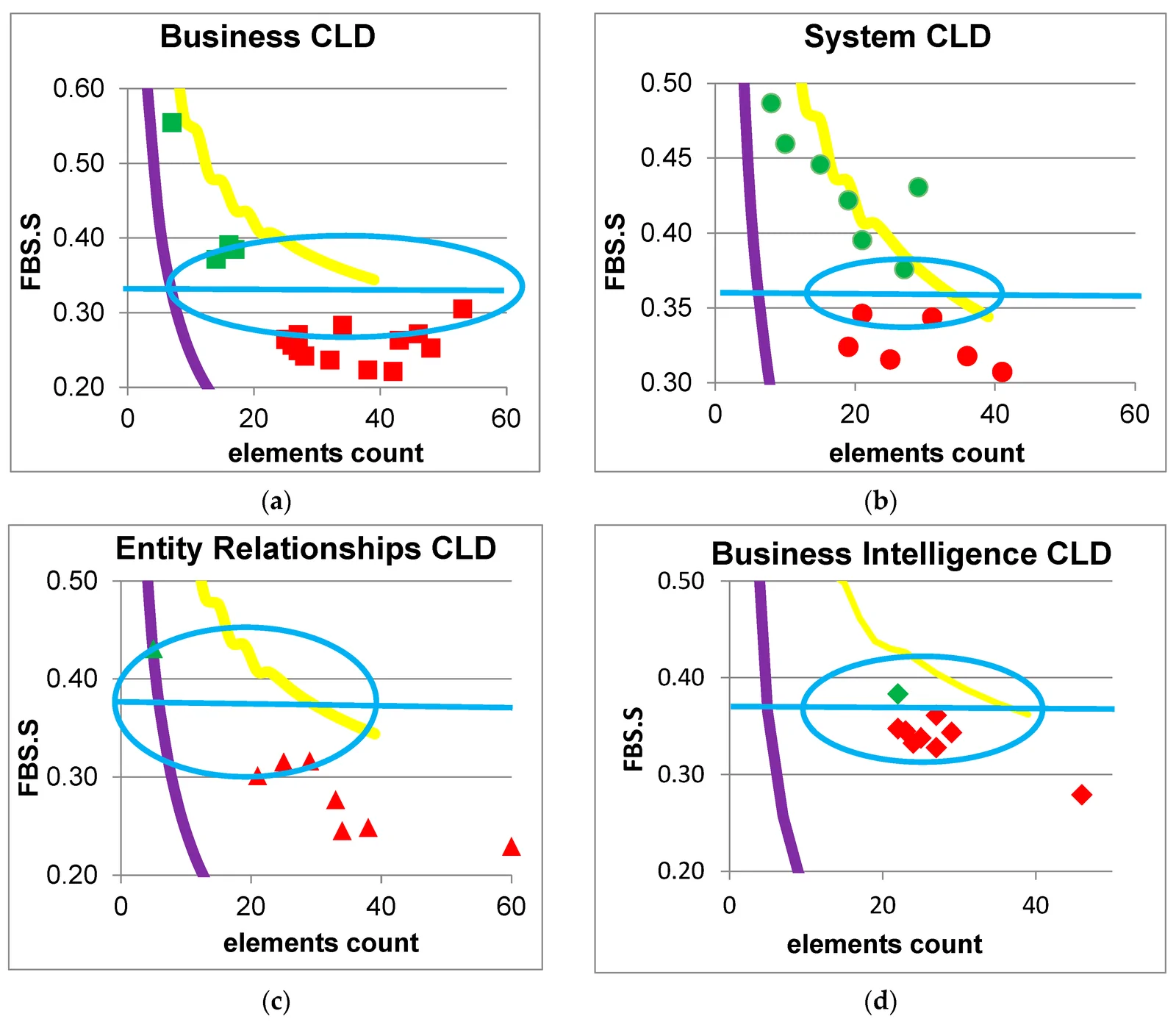
FBS26 metric values of the most numerous studied industrial CLDs in the set of 123 studied diagrams: (**a**) business CLDs, (**b**) system CLDs, (**c**) entity-relationship (ER) CLDs, and (**d**) business-intelligence CLDs. The horizontal line indicates the proposed boundary between mature and immature diagrams. Blue ellipses indicate diagrams located close to the boundary and, therefore, require additional inspection.

Figure 4 shows the distribution of industrial UML class diagrams by diagram category. The horizontal line in each plot represents the proposed empirical boundary between structurally mature and immature diagrams. Users may define alternative thresholds depending on the intended application and dataset. Diagrams located close to the boundary should be treated as borderline cases and may require additional expert inspection; these cases are indicated by ellipses.

Table 5 and Figure 4 show the following observations:

- immature Business CLDs (less than 18 elements) have FBS26.S metric values greater than 0.371; thus, we can assume the border between mature and immature Business CLDs to be 0.34;
- mature System CLDs (from 19 to 59 elements and having all kinds of elements) have FBS26.S metric values less than 0.347;
- immature System CLDs (less than 28 elements) have FBS26.S metric values greater than 0.377; thus, we can assume the border between mature and immature System CLDs to be 0.36;
- mature ER CLDs (from 21 to 98 elements) have FBS26.S metric values less than 0.318;
- immature ER CLDs (less than 6 elements) have FBS26.S metric values greater than 0.43; thus, we can assume the border between mature and immature ER CLDs to be 0.37;
- mature BI CLDs (from 22 to 145 elements) have FBS26.S metric values less than 0.362;
- immature BI CLDs (less than 23 elements) have FBS26.S metric values greater than 0.384; thus, we can assume the border between mature and immature BI CLDs to be 0.37;
- for conceptual and vocabulary CLDs, there is insufficient data to establish appropriate thresholds for maturity and immaturity; thus, we assumed them all to be mature.

The results show differences between diagrams labeled as mature and immature within the evaluated diagram categories. Diagrams regarded by practitioners as mature occupy relatively stable entropy ranges, whereas immature diagrams frequently appear outside these intervals. In several cases, the metric also identified diagrams requiring additional expert inspection, indicating that entropy-based assessment may provide useful decision support during architectural reviews. These observations indicate that FBS26 can serve as an architectural maturity indicator rather than merely a measure of information content.

To complement the controlled experiments on artificial diagrams, the study also considered a repository of 18 software projects [13]. The repository provides a broader empirical context for examining FBS26’s behavior across different UML modeling environments.

An additional observation concerns concentration effects. Diagrams containing large numbers of identical UML element kinds frequently produced substantially different entropy values from diagrams exhibiting a more balanced distribution of modeling constructs, even when both contained similar numbers of elements. This confirms that FBS26 captures structural characteristics that remain invisible to traditional size-based metrics.

Overall, the combination of controlled artificial experiments and substantial industrial validation demonstrates that systematic calibration substantially improves entropy-based measurement while preserving consistent behavior across diverse UML modeling environments. The results suggest that FBS26 may serve as a systematic indicator for assessing UML class diagram maturity during software development.

### 4.4. Threats to Validity

As with any empirical study, the results presented in this paper are subject to several threats to validity. Following common practice in empirical software engineering, these threats are discussed with respect to construct, internal, external, and conclusion validity.

- Construct Validity

The primary threat to construct validity concerns the interpretation of information content in UML class diagrams. FBS26 evaluates the distribution of UML element kinds using normalized Shannon entropy and calibrated element weights. Consequently, the metric reflects the structural information contained in a diagram rather than its semantic correctness or domain-specific meaning. Two diagrams with similar structural characteristics but different semantic content may therefore obtain similar metric values.

Another potential threat arises from the selected UML element weights. Although the proposed calibration procedure replaces subjective expert judgment with a systematic optimization process, the resulting weights are still derived from a predefined set of artificial calibration diagrams. Alternative calibration datasets could potentially produce slightly different weight values.

- Internal Validity

Internal validity concerns whether the observed improvements can genuinely be attributed to the proposed calibration procedure. To minimize this threat, FBS26 was evaluated using the same artificial diagram series that were previously employed in the analysis of FBS16. Consequently, improvements in entropy behavior result exclusively from the modified weighting scheme rather than from changes in the experimental design or entropy formulation.

The industrial evaluation was performed using a consistent implementation of the metric and identical processing procedures for all analyzed UML models, reducing the likelihood of implementation-related bias.

- External Validity

The primary threat to external validity concerns the representativeness of the UML models analyzed. The empirical validation dataset contains 123 UML class diagrams from four industrial software projects selected from a repository of 18 projects. These four projects were purposively selected because they contain relatively large numbers of class diagrams. Consequently, the dataset should not be interpreted as statistically representative of the complete repository, and the proposed maturity thresholds should not be interpreted as universal values applicable to every software project.

Furthermore, the present study focuses exclusively on UML class diagrams. Additional investigations are required to determine whether the proposed calibration procedure can be generalized to other UML diagram types, such as sequence, activity, state machine, or component diagrams.

- Conclusion Validity

The conclusions drawn in this paper are based on two complementary forms of evaluation: controlled experiments using artificial UML diagram series and empirical validation using a substantial industrial validation dataset. This combination reduces the likelihood that the observed improvements are artifacts of a particular experimental setting. Accordingly, maturity in this study refers to architectural maturity of UML class diagrams rather than semantic completeness or design correctness.

Nevertheless, the study does not claim that FBS26 completely replaces expert assessment. Instead, the metric should be viewed as a systematic quantitative indicator to help software architects evaluate diagrams. Additional replication studies involving independent industrial repositories and publicly available UML datasets would further strengthen confidence in the proposed approach and improve the generalizability of the reported results.

The benchmark diagrams and calibration procedure are intended to provide a reproducible reference framework for future comparisons of entropy-based UML metrics.

- Threshold Selection Validity

The proposed maturity thresholds are not statistically optimized boundaries, but expert-validated reference values derived from 123 class diagrams across 18 projects. Initial maturity classifications by an author—based on architecture, abstraction, and experience—were validated by project reviewers during implementation. Subsequent calculation of FBS26.S values aligned these classifications with a specific diagram type. Consequently, these values serve as context-specific practical guidelines rather than rigid rules, allowing other teams to adopt, adapt, or redefine them.

## 5. Discussion and Conclusions

This study investigated the problem of measuring information content in UML class diagrams using normalized entropy. While previous research demonstrated that entropy-based measures can provide meaningful insights into software models, the influence of UML element weighting schemes remained insufficiently understood. The work presented in this paper addresses this issue by introducing a systematic and reproducible weight-calibration procedure and incorporating it into FBS26. Accordingly, FBS26 should be interpreted primarily as an architectural maturity metric, while completeness and consistency provide complementary insights into how information is distributed across the three architectural dimensions.

The experimental results suggest that the proposed approach improves several aspects of metric behavior. In particular, it reduces the occurrence of false lack-of-information cases and decreases non-monotonic changes in metric values. These improvements increase the metric’s interpretability and make comparisons between diagrams more reliable.

The industrial evaluation demonstrates that FBS26 may be viewed as an architectural maturity metric for UML class diagrams, where maturity is interpreted through the amount and distribution of architectural information represented in the model. Although the metric should not be viewed as a replacement for architectural expertise, it can indicate which diagrams require additional attention. This may be especially valuable in large projects where manual inspection of all models is impractical.

In particular, it is worth noting that the observed distributions of FBS26.S values suggest that the metric may also support the identification of commonly distinguished UML class-diagram categories, including conceptual, business, and system class diagrams; entity-relationship diagrams; business intelligence table diagrams; and domain dictionary diagrams. This type of application of the FBS26 metric can be broadly applicable to a wide range of IT projects.

At the same time, the study has several limitations. The metric remains a syntactic measure and does not consider the semantics of class names, attributes, operations, or relationships. Consequently, two diagrams with identical structural characteristics may receive similar metric values even if their semantic content differs substantially. Furthermore, the validation focused exclusively on UML class diagrams, and additional studies will be required to determine whether the proposed calibration procedure can be generalized to other UML views.

Despite these limitations, the results demonstrate that systematic calibration of UML element weights can significantly improve entropy-based measurements. The findings, therefore, contribute to both the theory of software measurement and the practical assessment of UML models. The proposed metric, therefore, provides a quantitative measure of UML class diagram maturity by estimating its architectural information content, while completeness and consistency remain auxiliary characteristics describing the internal balance of that information.

Future research will focus on extending the calibration procedure to the functional and behavioral dimensions of UML models. Another promising direction is incorporating semantic information into entropy calculations, which may further improve the metric’s ability to assess diagram quality and completeness. Finally, replication studies using publicly available UML repositories would provide additional evidence regarding the generalizability of the proposed approach. Future work will investigate architectural maturity assessment for other UML diagram types using the same entropy-based calibration framework.

## Figures and Tables

**Table 1:** Comparison of representative UML metric categories.

Category	What Is Measured	Representative Examples	Main Limitation
Size-based	Number of UML elements	Marchesi [18], Genero [19], CK-derived UML metrics [20]	Does not directly characterize information content
Complexity-based	Coupling, cohesion, inheritance, abstraction, maintainability	CK [20], QMOOD [21], structural complexity Zhou [27] (uses entropy distance)	Focuses primarily on structural complexity
Entropy-based	Distribution and diversity of UML elements	Harrison [25], Yi and Wu [11], Yi [26], FBS16 [12], Niepostyn and Daszczuk [10]	Metric values may depend on assumptions concerning element weighting

**Table 2:** Exemplary FBS.S metric values for CLD depending on the number of elements (UML element weights for the structure dimension). The set selected for FBS26 is shown on the gray background. Unneeded cases are given in red (false lack of information in set (1,1,1,1), non-monotony in set (3,1,1,5)).

	(1,1,1,1) Class = 1, Attribute = 1, Operation = 1, Association = 1				(5,3,1,7) Class = 5, Attribute = 3, Operation = 1, Association = 7				(3,1,1,5) Class = 3, Attribute = 2, Operation = 1, Association = 5				(3,2,2,5) Class = 3, Attribute = 2, Operation = 2, Association = 5			
#	ClAss	AssAt	Op	Diag	ClAss	AssAt	Op	Diag	ClAss	AssAt	Op	Diag	ClAss	AssAt	Op	Diag
3	0.579	1.000	0.579	1.000	0.617	0.950	0.545	0.950	0.627	0.937	0.613	0.937	0.627	0.937	0.622	0.937
5	0.418	0.655	0.311	0.828	0.430	0.615	0.427	0.710	0.430	0.596	0.424	0.744	0.430	0.596	0.364	0.789
7	0.351	0.516	0.211	0.695	0.356	0.480	0.354	0.651	0.353	0.463	0.327	0.678	0.353	0.463	0.257	0.705
9	0.313	0.439	0.159	0.623	0.315	0.406	0.303	0.540	0.311	0.391	0.267	0.560	0.311	0.391	0.199	0.592
11	0.287	0.390	0.127	0.573	0.288	0.359	0.265	0.525	0.284	0.298	0.225	0.518	0.284	0.345	0.161	0.567
13	0.269	0.355	0.106	0.537	0.269	0.326	0.236	0.467	0.264	0.313	0.195	0.482	0.264	0.313	0.136	0.509
15	0.255	0.329	0.090	0.509	0.254	0.301	0.213	0.462	0.250	0.246	0.172	0.453	0.250	0.290	0.117	0.498
17	0.244	0.309	0.079	0.488	0.243	0.282	0.194	0.425	0.238	0.271	0.154	0.437	0.238	0.271	0.103	0.462
19	0.235	0.293	0.070	0.469	0.233	0.267	0.178	0.422	0.229	0.215	0.139	0.413	0.229	0.257	0.092	0.456
21	0.227	0.280	0.063	0.454	0.225	0.254	0.164	0.396	0.221	0.204	0.127	0.383	0.221	0.245	0.083	0.430
23	0.221	0.268	0.057	0.441	0.219	0.243	0.153	0.395	0.214	0.194	0.117	0.385	0.214	0.235	0.076	0.426
31	0.202	0.237	0.041	0.403	0.199	0.214	0.119	0.359	0.195	0.206	0.089	0.369	0.195	0.206	0.056	0.387
39	0.189	0.217	0.033	0.378	0.186	0.195	0.098	0.335	0.182	0.189	0.071	0.344	0.182	0.189	0.044	0.362

**Table 3:** Element kind weights in functionality, behavior, and structure dimensions (F,B,S) in example experiments. Only the S dimension is used in our analysis, according to the UML standard (structure diagrams).

Class	Attribute	Operation	Association
(0,0,1)	(0,0,1)	(1,1,1)	(0,0,1)
(0,0,3)	(0,0,1)	(1,1,1)	(0,0,1)
(0,0,3)	(0,0,1)	(1,1,1)	(0,0,2)
(0,0,3)	(0,0,1)	(1,1,1)	(0,0,5)
(0,0,3)	(0,0,2)	(1,1,1)	(0,0,5)
(0,0,3)	(0,0,2)	(1,1,2)	(0,0,1)
(0,0,3)	(0,0,2)	(1,1,2)	(0,0,5)
(0,0,5)	(0,0,3)	(1,1,1)	(0,0,7)

**Table 4:** Criteria for weight set choice.

Rank	Average Δ*_W_*_(3)_	Weight Set	Defect
1	−0.110	(1,1,1,1)	False lack of information
2	−0.100	(3,2,2,5)	
3	−0.093	(3,2,1,5)	
4	−0.088	(3,2,2,1)	
5	−0.086	(3,1,1,5)	non-monotonicity
6	−0.086	(5,3,1,7)	
7	−0.079	(3,1,1,2)	non-monotonicity
8	−0.066	(3,1,1,1)	

**Table 5:** Structural characteristics and FBS26 values of the industrial UML class diagrams. Architectural maturity is encoded as i (immature) and m (mature). Diagram types are C (conceptual), B (business), S (system), ER (entity-relationship), I (Business Intelligence), and V (vocabulary).

Diagram Symbol	Elements Count	Class	Attribute	Operation	Association	DirectedRelationship	FBS26.S	Maturity	Diagram Type	Diagram Symbol	Elements Count	Class	Attribute	Operation	Association	DirectedRelationship	FBS26.S	Maturity	Diagram Type
Conceptual Class diagrams										Class diagrams of system view									
eirz-22c	22	10	0	0	10	2	0.303	m	C	ecs-8	8	4	0	2	0	2	0.487	i	S
eirz-22d	22	11	0	0	11	0	0.214	m	C	ecs-10	10	3	0	4	0	3	0.460	i	S
swpl-80	80	34	0	0	38	8	0.214	m	C	ecs-27	27	6	16	0	4	1	0.376	i	S
swpl-40	40	20	0	0	1	19	0.213	m	C	ecs-21a	21	3	16	0	1	1	0.346	m	S
swpl-55	55	28	0	0	0	27	0.166	m	C	ecs-31	31	4	12	14	0	1	0.344	m	S
Class diagrams of business view										ecs-19b	19	3	14	0	2	0	0.324	m	S
swpl-7	7	2	4	0	1	0	0.555	i	B	ecs-36	36	6	25	0	4	1	0.318	m	S
ecs-16	16	4	9	0	3	0	0.391	i	B	ecs-25	25	5	17	0	3	0	0.316	m	S
ecs-17	17	5	8	0	4	0	0.385	i	B	ecs-41	41	7	28	0	5	1	0.307	m	S
swpl-14	14	2	10	0	2	0	0.372	i	B	ecs-38	38	4	31	0	3	0	0.226	m	S
nik-53	53	10	34	0	7	2	0.305	m	B	ecs-59	59	8	48	0	3	0	0.195	m	S
swpl-34	34	3	27	0	3	1	0.283	m	B	ER Class diagrams									
ecs-46	46	9	30	0	7	0	0.272	m	B	ecs-5	5	2	3	0	0	0	0.431	i	E
swpl-27a	27	8	18	0	1	0	0.271	m	B	ecs-29	29	6	17	0	6	0	0.317	m	E
swpl-25	25	3	20	0	2	0	0.264	m	B	ecs-25a	25	5	17	0	3	0	0.316	m	E
swpl-43	43	4	34	0	4	1	0.263	m	B	ecs-21c	21	3	16	0	2	0	0.302	m	E
swpl-26	26	3	21	0	2	0	0.257	m	B	ecs-98	98	5	88	0	5	0	0.131	m	E
swpl-48	48	4	38	0	5	1	0.253	m	B	ecs-43	43	2	40	0	1	0	0.120	m	E
swpl-27b	27	3	22	0	2	0	0.249	m	B	Business Intelligence Class diagrams									
nik-70	70	13	44	0	13	0	0.247	m	B	eirz-22e	22	4	15	0	1	2	0.383	i	I
nik-89	89	9	59	0	19	2	0.243	m	B	eirz-14a	27	4	19	0	2	2	0.361	m	I
swpl-28	28	3	23	0	2	0	0.242	m	B	eirz-22a	22	5	13	0	4	0	0.347	m	I
nik-128	128	20	77	0	28	3	0.239	m	B	eirz-139	132	13	100	0	17	2	0.207	m	I
swpl-32	32	7	24	0	1	0	0.237	m	B	eirz-145	145	22	96	0	27	0	0.204	m	I
swpl-11	11	2	9	0	0	0	0.235	m	B	eirz-84	84	13	70	0	1	0	0.143	m	I
nik-66	66	9	49	0	8	0	0.227	m	B	Vocabulary Class diagrams									
swpl-144	144	15	102	0	5	22	0.226	m	B	ecs-38a	38	4	22	12	0	0	0.231	m	V
swpl-38	38	9	28	0	1	0	0.224	m	B	ecs-50	50	4	34	12	0	0	0.186	m	V
swpl-70	70	6	56	0	7	1	0.222	m	B	eirz-196	197	28	169	0	0	0	0.094	m	V
eirz-201	194	19	137	0	38	0	0.180	m	B										

## Data Availability

Repository of projects from which industrial CLDs were taken for the measure validation: https://github.com/Niejurek/FBS26/blob/main/ClassRepository.eapx (accessed on 1 October 2026).

## Abbreviations

The following abbreviations are used in this manuscript:

UML	Unified Modeling Language
FBS	Information content measure in Functionality, Behavior, and Structure views
CLD	Class Diagram
FLI	Number of False Lack-of-Information
ANMV	Average Number of Monotonicity Violations
DMBD	Decrease In Metric for Balanced Diagrams
